# Intestinal carriage of antibiotic resistant *Acinetobacter baumannii* among newborns hospitalized in Moroccan neonatal intensive care unit

**DOI:** 10.1371/journal.pone.0209425

**Published:** 2019-01-10

**Authors:** Btissam Arhoune, Bouchra Oumokhtar, Fouzia Hmami, Samira El Fakir, Kaoutar Moutaouakkil, Fouzia Chami, Abdelhak Bouharrou

**Affiliations:** 1 Laboratory of Microbiology and Molecular biology, Faculty of Medicine and Pharmacy, Sidi Mohammed Ben Abdellah University, Fez, Morocco; 2 Laboratory of Biotechnology, Faculty of Sciences Dhar El Mahraz, Sidi Mohammed Ben Abdellah University, Fez, Morocco; 3 Neonatal Intensive Care Unit, University Hospital Hassan II, Fez, Morocco; 4 Laboratory of Epidemiology and Clinical Research, Faculty of Medicine and Pharmacy, Sidi Mohammed Ben Abdellah University, Fez, Morocco; St George’s University of London, UNITED KINGDOM

## Abstract

This study was conducted in order to assess the acquisition rate of *Acinetobacter baumannii* by newborn screening, on admission and during the discharge process of neonatal intensive care unit. (NICU). Furthermore, we investigated risk factors for potential colonization and molecular epidemiology of isolated resistant bacteria. This prospective study was conducted in the neonatal unit of Hassan II University Hospital of Fez from February 2013 to July 2015. During this period, all consecutive admitted neonates were screened for *A*. *baumannii* intestinal carriage, on admission and during the discharge process. Bacteriological and molecular tests were evaluated according to the international standards. This study examines the screening on admission of 455 newborns, 59% of whom were male. The average gestational age and birth weight were 35.2 weeks and 2612.1 g respectively. In total, 277 patients were included in the acquisition study on admission. The prevalence of multi-drug resistant (MDR) *A*. *baumannii* strain carriage was 6.5%, while the acquisition rate during the hospital recovery was 13.7%. In this study, 68 MDR *A*. *baumannii* isolates were collected. The resistance rates to different antibiotic classes including, Ceftazidime, Gentamycin and Ciprofloxacin varied between 92 and 100%. Moreover, 13% of MDR *A*. *baumannii* isolates were carbapenemase producers and 88% harbored *bla*_*OXA-23*_ gene. On admission, three risk factors were significantly associated with *A*. *baumannii* colonization: age (OR, 2.803; IC95%, 1.191–6.596; P = 0.01), gender (OR, 0.382; IC95%, 0.158–0.921; P = 0.03) and the delivery birth at the Maternity of University Hospital (MUH), (OR, 0.196; IC95%, 0.071–0.540; P = 0.002). However during hospitalization, the only risk factor associated with acquisition of *A*. *baumannii* was the respiratory distress (OR, 2.270; IC95%, 1.055–4.881; P = 0.03). A high intestinal carriage rate of *A*. *baumannii* and multiple antibiotic resistance were found in our NICU. Thus, the spread of MDR *A*. *baumannii* should be monitored by an active surveillance strategy.

## Introduction

*Acinetobacter baumannii* has been established as one of the leading nosocomial pathogens worldwide. It causes a spectrum of diseases which affects the following areas: the respiratory tract, the bloodstream, the surgical site, wound infections and the urinary tract [1]. An increasing number of nosocomial infections caused by this pathogen, particularly bacteremia and pneumonia were noted worldwide among patients admitted to Intensive Care Units (ICUs) [2]. Such infections might be associated with considerably increased mortality rates (52% and 34.1% respectively) [3,4].

*A*.*baumannii* is one of the most difficult acquired pathogens to control at ICUs because of both its ability to survive in hospital environments and its capacity ofacquiring genes resistance rapidly by different mechanisms including plasmids, transposons and integrons’ acquisition [5]. The most common antimicrobial resistance reported in *A*. *baumannii* is towards carbapenem [6]. The acquired carbapenem resistance is often associated with the OXA-type carbapenemases and metallo-β-lactamases [7]. This bacterium has also an intrinsic production of beta-lactamases with carbapenemases properties [8].

Episodes of *A*. *baumannii* infection have been reported as clustered epidemics with contamination of environmental sources or transmission from hand to hand of health care workers, which ultimately lead to colonization or subsequent infection of patients [9]. Different mechanisms of MDR *A*. *baumannii* acquisition were reported such as cross-transmissions, comorbidities, antibiotic treatment duration including carbapenems therapy, environment and severity of acute illness [8]. Moreover, prior colonization with *A*.*baumannii* has been found to be a risk factor for neonatal infections [10].

As is the case of a lot of countries, this bacterium is frequently isolated in Moroccan hospitals [11]. This study is the first of its kind to highlight the intestinal MDR *A*. *baumannii* carriage and its resistance to antibiotics as seen in newborns in Moroccan NICU. Therefore, we performed this prospective observational study to determine the prevalence of nosocomial acquisition of multidrug-resistant *A*. *baumannii* intestinal carriage amongst neonates in Moroccan NICU. Carriage rate at admission, risk factors of colonization, resistance profiles and genotypic characteristics were also studied.

## Methods

### Study design

This prospective study was conducted at the service of neonatology and intensive care unit of the University Hospital of Fez (Morocco). The hospital setting is a medical and surgical NICU that has 18 beds divided into 2 sectors (9 beds for each one); sector 1 corresponds to an intensive care unit and sector 2 corresponds to a preterm baby unit. This NICU is the only one in Fez, a city with an estimated population of approximately 1.5 million inhabitants. Despite the fact that the hand-hygiene compliance was not monitored in our NICU, standard hygiene precautions were nevertheless respected, such as hand hygiene before and after each contact with a patient and the surrounding surfaces, and contact isolation precautions with gloves for proven cases of carriage with *A*. *baumannii* as well as for patients colonized with multi-drug-resistant bacteria. The most common empiric antibiotic regimen in case of clinical suspicion of nosocomial sepsis was imipenem for the coverage of *A*. *baumannii*.

### Patient’s selection

The patient’s selection is from February 2013 to July 2015 and, all consecutive neonates admitted into the unit are also included. Only the first NICU admission per neonate was examined in the analysis. Babies were evaluated for *Acinetobacter baumannii* intestinal carriage at admission (a) and *Acinetobacter baumannii* acquisition during hospitalization (b). Imported carriers were excluded from the acquisition analysis to take into account just babies who acquired *A*. *baumannii* during their NICU stay. Furthermore, those without follow-up samples (due to death or discharge before the scheduled follow-up sampling) were excluded.

## Ethical approval

This study was approved by the Joint Research Ethics Committee of Medical School and university Hospital Hassan II of Fez (Fez, Morocco). Written information about the nature of the experimental procedures was given to parents of patients, who were asked for their consent.

### Statistical analysis

Potential risk factors associated with *A*. *baumannii* colonization were studied. The socio-demographic and clinical characteristics of patients were collected prospectively using a standard written questionnaire. Statistical analysis was carried out using SPSS, version 20 (SPSS Inc., Chicago, IL, USA) software. Results for quantitative variables were presented as mean ± standard deviation and for qualitative variables as number (percentage). Then, an univariate analysis was performed to establish all associations between gender, age, birth weight, prematurity, birthplace, admission and birth route, date of hospitalization, NICU admission and diagnosis after NICU admission. During NICU hospitalization, antimicrobial therapy, breastfeeding, central or peripheral venous catheterization and length of hospital stay were also recorded in the questionnaire. Chi-square test and Fisher’s exact test were used to established significant association when appropriate. The *P* < 0.05 was deemed as statistically significant. The multivariate analysis was performed to identify a potential risk factor associated with intestinal MDR *A*. *baumannii* colonization using simple logistic regression analysis. All variables with p<0.2 in an univariate analysis were included in a logistic regression model for a multivariate analysis. Odds ratios were presented with the corresponding 95% confidence intervals (OR, CI 95%).

### Sampling and screening

Two rectal swabs were collected from each newborn. The initial sample was performed up to 6 hours from admission to the NICU and the second one after 5 days of hospitalization in order not to lose patients in this schedule screening. Rectal swab specimens were enriched in nutrient broth BHI (Brain Heart infusion, Oxoid) at 37°C for 24h. Then, they were inoculated on Mac Conkey agar plates and incubated at 37°C for 24h. The identification of *A*. *baumannii* isolates was performed by classical bacteriological techniques (Gram stain, Oxidase test and Fermentation Glucose test) and confirmed by using API 20 NE galleries (Biomérieux, Marcy l’Etoile, France). Strains were originally identified as *Acinetobacter baumannii-calcoaceticus* complex and *A*. *baumannii* species confirmation was performed by *bla*_*OXA-51*_ gene PCR amplification.

### Antimicrobial susceptibility testing

As recommended by the EUCAST 2013, the following antimicrobial agents (Oxoid) were tested to evaluate susceptibility by disk diffusion method: Ticarcillin TIC (75 μg), Ceftazidime CAZ (30μg), Piperacillin PEP (75μg), Piperacillin/Tazobactam PTZ (75/10μg), Imipenem IMP (10μg), Gentamicin GN (10μg), Amikacin AK (30μg), Tobramycin TOB (10μg), Ciprofloxacin CIP (5 μg) and Trimethoprim/Sulfamethoxazole SXT (1.25/23.75μg). Isolates of resistant *A*. *baumannii* to three or more classes of antibiotics were considered as MDR (6) and those ones with an inhibition zone <17mm were treated as resistant to imipenem. The modified Hodge test and the Ethylene-Diamine-Tetra-Acetic (EDTA) disk synergy test were performed to screen for carbapenemase production. A difference of inhibition zone diameter >5 mm between imipenem disks and imipenem plus EDTA was interpreted as metallo-β-lactamase MBL positive. All resistant strains to Imipenem were screened by conventional single-plex PCR assay for the following carbapenemases encoding genes: *bla*_*OXA-23*,_ *bla*_*OXA-24*,_ *bla*_*OXA-51*,_ *bla*_*OXA-58*,_ *bla*_*KPC*,_ *bla*_*NDM*,_ *bla*_*IMP*_ and *bla*_*VIM*_.

### Preparation of DNA template for PCR

The total DNA was extracted by suspending few colonies of an overnight culture of *A*. *baumannii* isolates in 500 μL of DNase- and RNase-free water (Invitrogen, Paisley, UK). The suspension was boiled at 100°C for 10 min in a thermal block (Polystat 5, Bioblock Scientific, France), then centrifuged at 14000 x *g* for 10 min. An aliquot of 2 μL of the supernatant was used as a DNA template for PCR.

### Detection of carbapenemases encoding genes

Amplification reactions to detect carbapenemases encoding genes were performed in a volume of 50 μL containing, 2 μL of DNA template, 2.5mM MgCl_2_, 0.4μM of each forward and reverse primers, 100 μM of each dNTP, and 2 units *Taq* DNA polymerase (Promega, Madison, USA) in 1X PCR buffer provided by the manufacturer’s instructions. The amplification conditions were described previously [12,13]. Known carbapenemase producing strains were used as positive controls. PCR products were detected on 1% agarose gel (FMC Bioproduct, Rockland, ME) after ethidium bromide staining, UV illumination and photographed by an Olympus digital camera (Olympus Soft Imaging Solutions GmbH, Münster, Germany).

## Results

During the study period, 455neonates were screened. The average gestational age and mean birth weight were 35.2 (±3.2) weeks and 2612.1 g (±1023.2) respectively.

Of 455 patients screened for *A*. *baumannii*, 45 were carriers at NICU admission (9.8%). These patients were excluded from acquisition analysis. The remaining 90.2% (410 babies) were evaluated for *A*. *baumannii* acquisition. Of these, 133 (32.4%) patients were excluded, as they had no follow-up samples: 94 were discharged and 39 died before the planned sampling.

Finally, 277/455 (60.8%) were evaluated for *A*. *baumannii* acquisition. Twenty-nine (13.7%) out of 277 newborns had acquired *A*. *baumannii* in our NICU with a mean age of 9±7.2 days (S.D. Standard Deviation).

### *Baumannii* intestinal carriage

On the day of admission, the prevalence of *A*. *baumannii* rectal carriage was 9.8% (45/455), 30 babies had MDR *A*. *baumannii* (6.5%) and 6 had Carbapenemase producing (CP) *A*. *baumannii* (1.3%). Overall admission carriers, 68% were males (31/45), 71% were <48h old (32/45), 75% were born in the UH maternity unit (34/45), 62% came directly from the UH maternity unit to NICU (28/45) and 53.5% were < 24h old [Table 1].

**Table 1:** Association between patient’s characteristics and prevalence of multidrug-resistant *A*.*baumannii* carriage at the day of admission and during hospitalization at NICU.

	At admission				During NICU stay			
Category	Patients numbers (%) n = 455	MDR-AB- [N (%)]	MDR-AB+ [N (%)]	p-value	Patients numbers (%) n = 277	MDR-AB - [N (%)]	MDR-AB+ [N (%)]	p-value
Gender								
Male	267(58.7)	244 (57.4)	23 (76.7)	**0.028**	162(58.5)	139(58.2)	23(60.5)	0.783
Female	188(41.3)	181 (42.6)	7 (23.3)		115(41.5)	100(41.8)	15(39.5)	
Age (days)								
0–2	Mean±S.D. 6.5±15.8 days	290 (68.2)	18 (60)	0.200	Mean±S.D 6.2±15 days	155(64.9)	32(84.2)	**0.018**
> 2		135 (31.8)	12 (40)			84(35.1)	6(15.8)	
Prematurity								
Yes	223(49)	205 (48.2)	18 (60)	0.145	142(51.3)	117(49)	25(65.8)	0.054
No	232(51)	220 (51.7)	12 (40)		135(48.7)	122(51)	13(34.2)	
Birth weight (g)								
< 2500	Mean±S.D. 2612±1023g	207 (48.7)	19 (63.3)	0.087	Mean±SD 2542±1020g	116(48.5)	24(63.2)	0.094
≥ 2500		218 (51.3)	11 (36.7)			123(51.5)	14(36.8)	
Pathology*								
Respiratory distress	259(56.9)	240 (52.7)	19 (63.3)	0.296	160(57.8)	132(55.2)	28(73.7)	**0.032**
Icterus	38(8.4)	35 (8.2)	3 (10)	0.467	22(7.9)	21(8.8)	1(2.6)	0.192
Surgical pathology	31(6.8)	31 (7.3)	0 (0)	0.112	21(7.6)	19(7.9)	2(5.3)	0.561
Neonatal suffering	34(7.5)	32 (7.5)	2 (6.7)	0.608	18(6.5)	16(6.7)	2(5.3)	0.740
Neonatal infections	35(7.7)	31 (7.3)	4 (13.3)	0.191	19(6.5)	16(6.7)	3(7.9)	0.786
Neurological distress	44(9.7)	42 (9.9)	2 (6.7)	0.428	30(10.8)	26(10.9)	4(10.5)	0.948
Congenital malformations	11(2.4)	11 (2.6)	0 (0)	0.468	6(2.2)	6(2.5)	0(0)	0.323
Others	49(11.8)	47 (11.1)	2 (6.7)	0.351	24(8.7)	22(9.2)	2(5.3)	0.422
Birthplace								
Maternity of UH Fez	265(58.2)	241 (56.7)	24 (80)	**0.043**	152(54.9)	128(53.6)	24(63.2)	0.369
Other hospitals	164(36)	159 (37.4)	5 (16.7)		105(37.9)	92(38.5)	13(34.2)	
Home	26(5.7)	25 (5.9)	1 (3.3)		20(7.2)	19(7.9)	1(2.6)	
Admission route								
Maternity of UH Fez	236(51.8)	217 (51.1)	19 (63.3)	0.404	137(49.5)	112(46.9)	25(65.8)	**0.010**
Other hospitals	124(27.3)	119 (28)	5 (16.6)		83(30)	71(29.7)	12(31.6)	
Home	95(20.8)	89 (20.9)	6 (20)		57(20.6)	56(23.4)	1(2.5)	
Birth route								
Vaginal	310(68.1)	291 (68.5)	19 (63.3)	0.345	196(70.8)	174(72.8)	22(57.9)	0.061
Caesarean section	145(31.9)	134 (31.5)	11 (36.7)		81(92.2)	65(27.2)	16(42.1)	
Length of stay (days)								
< 3	--	--	--	--	Mean±S.D 8.9±7.2 days	17(7.1)	1(2.5)	0.298
≥ 3		--	--			222(92.9)	37(94.4)	
Venous catheterization								
Peripheral	--	--	--	--	272(98.2)	234(97.9)	38(100)	0.362
Central	--	--	--		5(1.8)	5(2.1)	0(0)	
Breastfeeding								
Breastfed newborn	--	--	--	--	97(35)	88(36.8)	9(23.7)	0.115
Diet newborn	--	--	--		180(65)	151(63.2)	29(76.3)	
Antibiotherapy								
Ceftriaxon+Gentamicin	--	--	--	--	166(59.9)	143(59.8)	23(60.5)	0.935
Amoxicillin+Gentamycin	--	--	--		87(31.4)	73(30.5)	14(36.8)	0.437

*Neonates may have more than one reason for hospitalization;

AB: *A*. *baumannii*

During NICU stay, the prevalence of *A*.*baumannii* intestinal acquisition was 14% (39/277). Thirty eight newborns had MDR *A*. *baumannii*(13.7%) and three had CP *A*. *baumannii*(1%). About 84% of babies acquired *A*.*baumannii* when they were < 48h old at the time of admission (32/39), 73% had respiratory distress as reason for hospitalization (28/39) and 65% were premature (25/39). The clinical characteristics of the patients are summarized in Table 1.

### Risk factors

Characteristics significantly found to be associated with MDR *A*. *baumannii* carriage at admission were the following: male gender (P = 0.028) and birthplace (P = 0.043). In fact, 24 carriers of MDR *A*. *baumannii* were born at the UH maternity unit (80%). 18 neonates of them came directly from the UH maternity unit to NICU. On the other hand, male gender was also associated with MDR *A*. *baumannii* carriage and the majority of carriers were male (76.7%).

Statistical analysis showed that there was a significant difference between newborns who had acquired and those who had not acquired MDR *A*. *baumannii* with regard to the age (p = 0.01), prematurity (p = 0.05), respiratory distress (p = 0.03) and admission route (p = 0.01) [Table 1].

In a multivariate analysis, gender (OR, 0.382; 95% CI, 0.158 to 0.921; P = 0.03), age at NICU admission (OR, 2.803; 95% CI, 1.191 to 6.596; P = 0.01) and birth in the UH maternity unit (OR, 0.196; 95% CI, 0.071 to 0.540; P = 0.002) were statistically associated with carriage at admission. Furthermore, the respiratory distress was the single factor found associated with acquisition of MDR *A*. *baumannii*(OR, 2.270; 95% CI, 1.055 to 4.881; P = 0.03)[Table 2].

**Table 2:** Multivariable analysis of MDR A. baumannii carriage at NICU admission and acquisition during hospitalization.

Variable	Multivariable analysis for *A*.*baumannii* carriage at NICU admission		Multivariable analysis for *A*. *baumannii* acquisition	
	OR (95%CI)	P value	OR (95%CI)	P value
**Gender**	0.382 (0,158–0.921)	0,032	-	-
**Age**	2,803 (1,191–6,596)	0,018	-	-
**Birth place**	0.196 (0,071–0,540)	0,002	-	-
**Respiratory distress**	-	-	2,270 (1,055–4.881)	0.036

### Antibiotic resistance of *A*. *baumannii* isolates

A total of 84 *A*. *baumannii* isolates were collected: 53.5% at admission (n = 45) and 46.4% at discharge (n = 39). The majority of *A*. *baumannii* isolates (admission and discharge combined isolates) showed an MDR phenotype (68/84). More than 92% of isolates were resistant to PEP, TIC, TCA, CAZ, TOB, GN and CIP (77/84). Resistance rate for PTZ, SXT, AK and IMP were lower (ranged from 3–36%). In addition, 72.6% of isolates share the same antimicrobial resistance profile (61/84) as being resistant to PEP, TIC, TCA, CAZ, TOB, GN and CIP. However, each strain could be phenotypically distinguished if we took into consideration its susceptibility to the other antimicrobials tested.

At admission, all MDR *A*. *baumannii* isolates were resistant to PEP, TIC, TOB and GN (100%). Most of them exhibited resistance to TCA, CAZ and CIP (96%). During hospitalization, all MDR *A*. *baumannii* acquired isolates were resistant to CAZ and PEP (100%). Resistance rates range between 92–96% for TIC, TCA, TOB, GN and CIP [Table 3].

**Table 3:** Susceptibility patterns of the 69 isolated MDR *A*. *baumannii* strains.

Antibiotics	Nr. of AB isolates [N (%)]		Nr. of MDR-AB isolates [N (%)]		Nr. of MDR-AB carriers [N (%)]	
	N = 84		N = 68			
	Admission N = 45	Discharge N = 39	Admission N = 30	Discharge N = 38	Admission N = 455	Discharge N = 277
Pepiracillin/Tazobactam	8 (17.7)	14 (35.8)	8 (26.6)	14 (36.8)	8 (1.7)	14 (5.04%)
Pepiracillin	30 (66.6)	38 (97.4)	30 (100)	38 (100)	30 (6.5)	38 (13.7)
Ticarcillin	30 (66.6)	36 (92.3)	30 (100)	36 (94.7)	30 (6.5)	36 (12.9)
Ticarcillin/Clavulanic Acid	29 (64.4)	36 (92.3)	29 (96.6)	36 (94.7)	29 (6.3)	36 (12.9)
Ceftazidim	29 (64.4)	38 (97.4)	29 (96.6)	38 (100)	29 (6.3)	38 (13.7)
Imipenem	6 (13.3)	3 (7.69)	6 (20)	3 (7.8)	6 (1.3)	3 (1)
Tobramycin	30 (66.6)	37 (94.8)	30 (100)	37 (97.3)	30 (6.5)	37 (13.3)
Amikacin	1 (2.2)	7 (17.9)	1 (3.3)	7 (18.4)	1 (0.2)	1 (0.3)
Gentamycin	30 (66.6)	37 (94.8)	30 (100)	37 (97.3)	30 (6.5)	30 (10.8)
Ciprofloxacin	29 (64.4)	35 (89.7)	29 (96.6)	35 (92.1)	29 (6.3)	35 (12.6)
Trimethoprim/Sulfamethoxazole	3 (6.6)	5 (12.8)	3 (10)	5 (13.1)	3 (0.6)	5 (1.8)

AB: *A*. *baumannii*

Concerning imipenem resistance, 13.3% of MDR isolates were imipenem resistant (9/68), 20.5% were intermediate (14/68) and 66.2% were susceptible (45/68).

The Hodge test was positive for all isolates resistant to imipenem. Similarly, tests with IMP and IMP/EDTA indicated the presence of an MBL in these isolates.

### Molecular analysis of CP *A*. *baumannii* isolates using PCR

Among all imipenem resistant isolates, the resistance genes *bla*_*OXA-23*,_ *bla*_*OXA-24*,_ *bla*_*OXA-51*,_ *bla*_*OXA-58*,_ *bla*_*KPC*,_ *bla*_*NDM*,_ *bla*_*IMP*_ *and bla*_*VIM*_ had been investigated. The PCR results showed that all our isolates (100%) were positive for *bla*_*OXA-51*_ gene(n = 84), confirming the identification of *A*.*baumannii* species. Besides, 8 out of 9 CP *A*. *baumannii* isolates were positive for *bla*_*OXA-23*_ encoding gene. However, no strain had *bla*_*OXA-24*_ and *bla*_*OXA-58*_ genes in its genome. Likewise, PCRs for *bla*_*KPC*_, *bla*_*IMP*_, *bla*_*VIM*_ and *bla*_*NDM*_ were negative.

## Discussion

Colonization by MDR *A*. *baumannii* in a hospital is a serious concern worldwide, mainly because of limited therapeutic options for treating infections caused by this resistant pathogen. It was listed as one of the six top-priority dangerous microorganisms by the *Infectious Diseases Society of America* (IDSA) [14].

To the best of our knowledge, this is the first study in Morocco to focus on MDR *A*. *baumannii* intestinal carriage among babies during hospitalization at the NICU. The prevalence rate of *A*.*baumannii* acquisition was 14% in our study and almost all of the isolates were MDR (97%). A previous study performed in 2011 in France showed that 11% of hospitalised patients acquired MDR *A*. *baumannii* [15]. On the other hand, the main finding of our study was the prevalence of neonates colonization on admission which was 9.8% with more than 66% of MDR (6.5%). Our prevalence was lower than a previous study made in Spain (25%) and similar to an American one (8.7%) [16,17]. Recent study in Taiwan, found just 0.2% of *A*. *baumannii* intestinal carriage from ICU admitted patients [18]. Furthermore, mouth/throat, skin and rectal swabs samples in a Turkish ICU revealed that 6.3% of adults hospitalised were colonized by *A*.*baumannii* on admission [19].

This study also proves that birth at a UH maternity unit is a risk factor for MDR *A*. *baumannii* carriage. However, insufficient incubators in our NICU can prolong the length of stay at the UH maternity unit, which could explain, in part the colonization by MDR *A*. *baumannii*. Also, the lack of hygienic practices during delivery and postnatal care can promote this colonization during the first week of baby’s life [20]. During hospitalization, respiratory distress was the single risk factor of MDR *A*. *baumannii* acquisition found (OR, 2.270; 95% CI, 1.055 to 4.881; P = 0.03). Cisneros-Herreros *et al*. [21] have reported that acute respiratory distress syndrome is a risk factor for *A*. *baumannii* nosocomial pneumonia in ICU. More than 80% of infected patients were associated with mechanical ventilation [22]. The long duration of the mechanical ventilation has been reported by Zhang *et al*. [23] as a risk factor.

Prematurity, early age and the admission route of babies were also reported as parameters that increase dramatically the risk of acquisition and/or infection by MDR *A*. *baumannii* in the NICU [24,25]. In our univariate analysis, these factors were significantly associated with acquisition of this bacterium. Likewise, the very low birth weight of babies in the NICUs increases the colonization risk with nosocomial *A*.*baumannii* strains [26]. This variable had a tendency to be more associated with case patients, but the values did not reach any statistical significance. The length of stay in the ICU was reported as a risk factor of acquisition of MDR *A*. *baumannii* in several previous studies [10,27], but it was not significant in ours. A possible reason might be that for fear of losing patients we performed the discharge screening within the space of 5 days.

A very high resistance rate to commonly used antibiotics such as third-generation cephalosporin or gentamicin has been observed among our isolates. The prevalence of intestinal colonization by imipenem resistant *A*. *baumannii* from our NICU was 1%. The same rate was reported in France and Turkey [28,29]. The prevalence of acquired CP *A*. *baumannii* reported by Playford EG *et al*. was more important (4.5%) [30]. The genotyping results of our CP *A*. *baumannii* isolates confirmed the *A*. *baumannii* species through the presence of the intrinsic encoding gene *bla*_*OXA-51*_ and across; thanks to this specific character, we can differentiate between *A*. *baumannii* and other species of *A*. *calcoaceticus-baumannii* complex [31]. This gene was detected in all our CP *A*. *baumannii* (100%) and needs to be regulated upstream by IS*Aba1* to provide resistance [32]. In other surveys, the prevalence of *bla*_*OXA-51*_ gene was ranged between 80–100% [33,34]. Then, *bla*_*OXA-23*_ encoding gene was present in 89% of our isolates (8/9). Moreover, it was the main gene responsible of CP *A*. *baumannii*. It is either located on the chromosome or on plasmids and associated with four different genetic structures, with the most frequent being transposons *Tn*2006 [35]. Besides, it was the most prevalent carbapenemase-encoding gene circulating in the Mediterranean region [36]. A previous Moroccan research study detected *bla*_*OXA-23*_ *bla*_*OXA-51*_, *bla*_*OXA-24*_ and *bla*_NDM_ [37]. In the present work, no metallo-β-lactamase genes (*bla*_*IMP*_, *bla*_*VIM*_ and *bla*_*NDM*_) were detected in any of the *A*. *baumannii* isolates. The prevalence of these genes is generally low within *A*. *baumannii* strains isolated from ICUs [38] or absent in *A*. *baumannii* intestinal carriage strains in ICUs [28,39,40]. The *bla*_*KPC*_ gene had not been detected either. This gene has been identified worldwide in *Enterobacteriaceae* and *Pseudomonas aeruginosa* isolates [41,42] and, to date, KPC-producing *A*. *baumannii* has been reported only in Puerto Rico [43].

Finally, the prevention of MDR *A*. *baumannii* colonization in newborns is clearly difficult. Screening on admission allows early detection and limits dissemination of these strains with application of appropriate control measures. Implementation of barrier precautions for patients presenting identified risk factors would probably be useful in reducing the cross-transmission between neonates who are the most likely to be colonized. As is the case in most developing countries, a low awareness of hand hygiene practices among health-care professionals was observed in our ward. This NICU is unique in the region of Fez and provides facilities to more than 1200 patients per year. But this ward also is suffering from a lack of sufficient medical staff including 3 seniors and 6 nurses for 18 beds. Moreover, barrier precautions are time consuming and our ward has only a few single rooms. This situation only amplifies the risk of transmission and dissemination of epidemic strains.

There are several limitations to the current study. Firstly, the moment of discharge screening can lead to biased estimates of the association between length of stay and MDR *A*. *baumannii* acquisition. Secondly, since active surveillance for *A*. *baumannii* was not consistent throughout the study period, all admitted patients may not have been included in this study. Lastly, the evaluation of the clonal relationship between the different *A*.*baumannii* isolates was not performed to confirm the role of cross-transmission of these bacteria between patients.

## Conclusion

This study showed high prevalence of *A*. *baumannii* intestinal carriage, multiple antibiotic resistance profiles and diversity of encoding genes in our NICU. This situation required development of antimicrobial stewardship initiatives and maintaining of antimicrobial resistance surveillance systems. Furthermore, the knowledge of risk factor profiles may lead to develop strategies of colonization prevention and subsequent invasive disease in high risk hospitalized neonates.

## Supporting information

S1 Dataset (XLSX)

## References

[1] Ahmed SS, Alp E, Doğanay M. Challenge of intensive care unit-acquired infections and Acinetobacter baumannii in developing countries. OA Crit Care. 2013;1(2):1–5.

[2] Falagas ME, Kopterides P. Risk factors for the isolation of multi-drug-resistant Acinetobacter baumannii and Pseudomonas aeruginosa: a systematic review of the literature. J Hosp Infect [Internet]. 2006 9 [cited 2018 Mar 22];64(1):7–15. Available from: http://www.ncbi.nlm.nih.gov/pubmed/16822583 16822583 10.1016/j.jhin.2006.04.015

[3] Dijkshoorn L, Nemec A, Seifert H. An increasing threat in hospitals: baumannii. 2007;5(december). 10.1038/nrmicro1789 18007677

[4] Park SY, Choo JW, Kwon SH, Yu SN, Lee EJ, Kim TH, et al Risk Factors for Mortality in Patients with Acinetobacter baumannii Bacteremia. 2013;45(3):325–30. 10.3947/ic.2013.45.3.325 PMC3848511 24396634

[5] Manchanda V, Sanchaita S. Multidrug Resistant Acinetobacter. 2010; (3). 10.4103/0974-777X.68538 PMC2946687 20927292

[6] Maragakis LL, Perl TM. Antimicrobial Resistance: *Acinetobacter baumannii*: Epidemiology, Antimicrobial Resistance, and Treatment Options. Clin Infect Dis [Internet]. 2008 4 15 [cited 2018 Mar 22];46(8):1254–63. Available from: http://www.ncbi.nlm.nih.gov/pubmed/18444865 18444865 10.1086/529198

[7] Potron A, Poirel L, Nordmann P. Ac ce p te ip t. Int J Antimicrob Agents [Internet]. 2015; Available from: 10.1016/j.ijantimicag.2015.03.001 25857949

[8] Elkalioubie A, Nseir S. Acquisition of carbapenem-resistant Acinetobacter baumannii in the intensive care unit: just a question of time? 2016;4(15):15–8. 10.21037/atm.2016.09.01 PMC5104629 27867970

[9] Wei H, Hsu Y, Lin H, Hsieh T, Yen T, Lin H. ScienceDirect Multidrug-resistant Acinetobacter baumannii infection among neonates in a neonatal intensive care unit at a medical center in central Taiwan. J Microbiol Immunol Infect [Internet]. 2015;48(5):531–9. Available from: 10.1016/j.jmii.2014.08.025 25442873

[10] Moghnieh R, Siblani L, Ghadban D, Mchad H El, Zeineddine R, Abdallah D, et al Extensively drug-resistant Acinetobacter baumannii in a Lebanese intensive care unit: risk factors for acquisition and determination of a colonization score. J Hosp Infect [Internet]. 2016;92(1):47–53. Available from: 10.1016/j.jhin.2015.10.007 26616413

[11] Ait el kadi M, Aghrouch M, Seffar M, El harti J, Bouklouze A, Cherrah Y, et al Prévalence des souches d’Acinetobacter baumannii et de Pseudomonas aeruginosa résistantes à l’imipénème par production de métallo-β-lactamases. Med Mal Infect. 2006;36(7):386–9. 10.1016/j.medmal.2006.05.003 16842953

[12] Woodford N, Ellington MJ, Coelho JM, Turton JF, Ward ME, Brown S, et al Multiplex PCR for genes encoding prevalent OXA carbapenemases in Acinetobacter spp. 2006;27:351–3. 10.1016/j.ijantimicag.2006.01.004 16564159

[13] Poirel L, Walsh TR, Cuvillier V, Nordmann P. Multiplex PCR for detection of acquired carbapenemase genes. Diagn Microbiol Infect Dis [Internet]. 2011;70(1):119–23. Available from: 10.1016/j.diagmicrobio.2010.12.002 21398074

[14] Boucher HW, Talbot GH, Bradley JS, Edwards JE, Gilbert D, Rice LB, et al Bad Bugs, No Drugs: No ESKAPE! An Update from the Infectious Diseases Society of America. Clin Infect Dis [Internet]. 2009 1 1 [cited 2018 Apr 2];48(1):1–12. Available from: http://www.ncbi.nlm.nih.gov/pubmed/19035777 19035777 10.1086/595011

[15] Nseir S, Blazejewski C, Lubret R, Wallet F, Courcol R, Durocher A. Risk of acquiring multidrug-resistant Gram-negative bacilli from prior room occupants in the intensive care unit. Clin Microbiol Infect [Internet]. 2011;17(8):1201–8. Available from: 10.1111/j.1469-0691.2010.03420.x 21054665

[16] Corbella X, Pujol M, Ayats J, Ardanuy C, Dominguez MA, Linares J, et al Relevance of Digestive Tract Colonization in the Epidemiology of Nosocomial Infections Due to Multiresistant Acinetobacter baumannii. 1996; (9411112). 10.1093/clinids/23.2.329 8842272

[17] Lortholary O, Fagon J, Hoi AB, Mahieu G, Control SI, Epidemiology H, et al Colonization by Acinetobacter baumanii in Intensive-Care-Unit Patients Laurent Gutmann Published by: Cambridge University Press on behalf of The Society for Healthcare Epidemiology of America Stable URL: http://www.jstor.org/stable/30143440 REFERENCES L. 2016;19(3):188–90. 10.1086/647793 9552188

[18] Thuy DB, Campbell J, Van N, Hoang M, Thi T, Trinh T, et al A one-year prospective study of colonization with antimicrobial-resistant organisms on admission to a Vietnamese intensive care unit. PLOS ONE | [Internet]. [cited 2017 Oct 1]; Available from: https://www.ncbi.nlm.nih.gov/pmc/articles/PMC5599024/pdf/pone.0184847.pdf 10.1371/journal.pone.0184847 PMC5599024 28910379

[19] IJAR—Indian Journal of Applied Research—The Colonization and Infection Relationship inHospitalized Patients at Intensive Care Unit [Internet]. [cited 2018 Mar 22]. https://www.worldwidejournals.com/indian-journal-of-applied-research-(IJAR)/file.php?val=September_2015_1492582508__178.pdf

[20] Zaidi AKM, Thaver D, Ali SA. Pathogens Associated With Sepsis in Newborns and Young Infants in Developing Countries. 2009;28(1):10–8. 10.1097/INF.0b013e3181958769 19106757

[21] Cisneros-Herreros JM, Garnacho-Montero J, Pachón-Ibáñez ME. [Nosocomial pneumonia due to Acinetobacter baumannii]. Enferm Infecc Microbiol Clin [Internet]. 2005 12 [cited 2017 Sep 8];23 Suppl 3:46–51. Available from: http://www.ncbi.nlm.nih.gov/pubmed/16854341 16854341 10.1157/13091220

[22] Nosocomial infections in medical intensive care units in the …: Critical Care Medicine. [cited 2017 Aug 1]; http://journals.lww.com/ccmjournal/pages/articleviewer.aspx?year=1999&issue=05000&article=00020&type=abstract

[23] Zhang D-S, Chen C, Zhou W, Chen J, Mu D-Z. [Pathogens and risk factors for ventilator-associated pneumonia in neonates]. Zhongguo Dang Dai Er Ke Za Zhi [Internet]. 2013 1 [cited 2017 Jul 31];15(1):14–8. Available from: http://www.ncbi.nlm.nih.gov/pubmed/23336161 23336161

[24] Zhou BIN, Liu X, Wu JIEBIN, Jin BAO, Zhang YANYAN. Clinical and microbiological profile of babies born with risk of neonatal sepsis. 2016;3621–5. 10.3892/etm.2016.3836 PMC5228283 28101156

[25] Ertugrul S, Aktar F, Yolbas I, Yilmaz A, Elbey B, Yildirim A, et al Risk Factors for Health Care-Associated Bloodstream Infections in a Neonatal Intensive Care Unit. 2016 [cited 2017 Oct 1];26(5). Available from: https://www.ncbi.nlm.nih.gov/pmc/articles/PMC5297258/pdf/ijp-26-05-5213.pdf 10.5812/ijp.5213 PMC5297258 28203330

[26] Moati A, Al K, Hakeem A, Jadba N El, Al AS, El IA. Nosocomial multidrug-resistant Acinetobacter baumannii in the neonatal intensive care unit in Gaza City, Palestine. 2009;623–8. 10.1016/j.ijid.2008.08.029 19144555

[27] Masse J, Elkalioubie A, Blazejewski C, Ledoux G, Wallet F, Poissy J, et al Colonization pressure as a risk factor of ICU-acquired multidrug resistant bacteria: a prospective observational study. Eur J Clin Microbiol Infect Dis [Internet]. 2017 5 20 [cited 2017 Oct 1];36(5):797–805. Available from: http://link.springer.com/10.1007/s10096-016-2863-x 28000030 10.1007/s10096-016-2863-x

[28] Armand-Lefèvre L, Angebault C, Barbier F, Hamelet E, Defrance G, Ruppé E, et al Emergence of imipenem-resistant gram-negative bacilli in intestinal flora of intensive care patients. Antimicrob Agents Chemother. 2013;57(3):1488–95. 10.1128/AAC.01823-12 23318796 PMC3591916

[29] Yeşilbağ Z, Çağatay AA, Karadeniz A, Başaran S, Orhun G, Özcan PE, et al Yoğun Bakım Birimindeki Hastaların Rektal Kolonizasyonu ile Hastane Enfeksiyonu Arasında Bir İlişki Var mı? Is There a Relationship Between Rectal Colonization and Nosocomial Infection of Patients in Intensive Care Unit? @BULLET Kabul Edil Tarihi [Internet]. 2014 [cited 2018 Feb 23]; (04). Available from: http://www.mikrobiyolbul.org/managete/fu_folder/2015-03/2015-49-3-327-339.pdf

[30] Playford EG, Craig JC, Iredell JR. Carbapenem-resistant Acinetobacter baumannii in intensive care unit patients: risk factors for acquisition, infection and their consequences. 2007;204–11. 10.1016/j.jhin.2006.11.010 17254667

[31] Nordmann P. aux carbapénèmes chez les bacilles à Gram négatif. 2010;26. 10.1051/medsci/20102611950 21106177

[32] Turton JF, Ward ME, Woodford N, Kaufmann ME, Pike R, Livermore DM, et al The role of ISAba1 in expression of OXA carbapenemase genes in Acinetobacter baumannii. FEMS Microbiol Lett [Internet]. 2006 5 [cited 2018 Mar 23];258(1):72–7. Available from: http://www.ncbi.nlm.nih.gov/pubmed/16630258 16630258 10.1111/j.1574-6968.2006.00195.x

[33] Ehlers MM, Hughes JM, Kock MM. Prevalence of Carbapenemases in Acinetobacter baumannii In: Antibiotic Resistant Bacteria—A Continuous Challenge in the New Millennium [Internet]. InTech; 2012 [cited 2018 Mar 22]. http://www.intechopen.com/books/antibiotic-resistant-bacteria-a-continuous-challenge-in-the-new-millennium/antibiotic-resistance-genes-in-acinetobacter-baumannii-isolates

[34] Feizabadi MM, Fathollahzadeh B, Taherikalani M, Rasoolinejad M, Sadeghifard N, Aligholi M, et al Antimicrobial susceptibility patterns and distribution of blaOXA genes among Acinetobacter spp. Isolated from patients at Tehran hospitals. Jpn J Infect Dis [Internet]. 2008 7 [cited 2018 Mar 23];61(4):274–8. Available from: http://www.ncbi.nlm.nih.gov/pubmed/18653968 18653968

[35] Carbapenemase O-, Mugnier PD, Poirel L, Naas T, Nordmann P. Worldwide Dissemination of the. 2010;16(1):1–6. 10.3201/eid1601.090852 PMC2874364 20031040

[36] Djahmi N, Dunyach-remy C, Pantel A, Dekhil M, Sotto A, Lavigne J. Epidemiology of Carbapenemase-Producing Enterobacteriaceae and Acinetobacter baumannii in Mediterranean Countries. 2014;2014. 10.1155/2014/305784 PMC4052623 24955354

[37] Uwingabiye J, Lemnouer A, Roca I, Alouane T, Frikh M, Belefquih B, et al Clonal diversity and detection of carbapenem resistance encoding genes among multidrug-resistant Acinetobacter baumannii isolates recovered from patients and environment in two intensive care units in a Moroccan hospital. Antimicrob Resist Infect Control [Internet]. 2017 [cited 2018 Apr 2];6 Available from: https://www.ncbi.nlm.nih.gov/pmc/articles/PMC5615474/pdf/13756_2017_Article_262.pdf 10.1186/s13756-017-0262-4 PMC5615474 28959441

[38] Safari M, Mozaffari Nejad AS, Bahador A, Jafari R, Alikhani MY. Prevalence of ESBL and MBL encoding genes in Acinetobacter baumannii strains isolated from patients of intensive care units (ICU). Saudi J Biol Sci [Internet]. 2015 7 1 [cited 2018 May 2];22(4):424–9. Available from: https://www.sciencedirect.com/science/article/pii/S1319562X15000054 26150748 10.1016/j.sjbs.2015.01.004 PMC4486466

[39] Hammami S, Dahdeh C, Mamlouk K, Ferjeni S, Maamar E, Hamzaoui Z, et al No Title. 2017;00(00):1–8. 10.1089/mdr.2016.0205 28099062

[40] Aljindan R, Bukharie H, Alomar A, Abdalhamid B. Prevalence of digestive tract colonization of carbapenem-resistant Acinetobacter baumannii in hospitals in Saudi Arabia. J Med Microbiol [Internet]. 2015 4 1 [cited 2018 Mar 27];64(Pt_4):400–6. Available from: http://jmm.microbiologyresearch.org/content/journal/jmm/10.1099/jmm.0.000033 25657302 10.1099/jmm.0.000033

[41] Nordmann P, Cuzon G, Naas T. The real threat of Klebsiella pneumoniae carbapenemase-producing bacteria. Lancet Infect Dis [Internet]. 2009 4 1 [cited 2018 Apr 30];9(4):228–36. Available from: https://www.sciencedirect.com/science/article/pii/S1473309909700544 19324295 10.1016/S1473-3099(09)70054-4

[42] Queenan AM, Bush K. Carbapenemases: the versatile beta-lactamases. Clin Microbiol Rev [Internet]. 2007 7 1 [cited 2018 Apr 30];20(3):440–58, table of contents. Available from: http://www.ncbi.nlm.nih.gov/pubmed/17630334 17630334 10.1128/CMR.00001-07 PMC1932750

[43] Robledo IE, Aquino EE, Santé MI, Santana JL, Otero DM, León CF, et al Detection of KPC in Acinetobacter spp. in Puerto Rico. Antimicrob Agents Chemother. 2010;54(3):1354–7. 10.1128/AAC.00899-09 20038618 PMC2825984

